# Evaluation of Baseline Diffusion Tensor Imaging Biomarkers in phase 2 multi‐center PROSPECT‐ALZ study

**DOI:** 10.1002/alz.092353

**Published:** 2025-01-09

**Authors:** Diana O Svaldi, Saima Rathore, Yun‐Ju Cheng, Leanne M Munsie, Adam S Fleisher, Mirek Brys, Sergey Shcherbinin

**Affiliations:** ^1^ Eli Lilly and Company, Indianapolis, IN USA

## Abstract

**Background:**

Diffusion Tensor Imaging (DTI) biomarkers are increasingly utilized in clinical trials of Alzheimer’s disease (AD). They have shown promise in detecting AD related changes in white‐matter (WM) integrity (Raghavan et al., 2020) and gray‐matter (GM) microstructure (Ofori et al., 2019). Moreover, DTI‐derived connectome has been shown to be related to tau progression (Vogel et al., 2020). Our study evaluated baseline GM microstructure and WM integrity measures in the PROSPECT‐ALZ trial (NCT05063539) of LY3372689, an O‐GlcNAcase (OGA) inhibitor.

**Methods:**

PROSPECT‐ALZ is a phase 2, randomized, double‐blind, multicenter, placebo‐controlled trial in participants with early symptomatic AD and presence of tau pathology measured using flortaucipir PET. Participants who received DTI scans (n = 223, age=73.40 (6.0), MMSE=25.6 (2.3), N female=124) were divided into groups based on visual assessment of flortaucipir images (Fleisher et al., 2021) (negative, n=56 vs positive, n = 144) and cognition (MMSE≤24, n = 81 vs MMSE>24 & MMSE≤29, n=133). Group differences in DTI scalars fractional‐anisotropy (FA), mean‐diffusivity (MD), and freewater‐fraction (FW) were evaluated in 16 GM regions and 9 WM tracts using ANCOVA (α = 0.05, LSmean±SE), adjusting for age and gender. FA and MD values were FW corrected (Conklin et al., CTAD 2023).

**Results:**

Visually positive participants exhibited reduced FA in the hippocampus relative to negative participants (0.289±0.006 vs. 0.322±0.010). Positive participants exhibited increased MD in the hippocampus (1.224E‐03±2.328E‐05 vs. 1.055E‐03±3.692E‐05), isthmus cingulate (9.324E‐04±1.971E‐05 vs 8.440E‐04±3.126E‐05), medial temporal cortex (9.751E‐04±1.801E‐05 vs 8.772E‐04±2.855E‐05), and whole temporal cortex (1.050E‐03±1.707E‐05 vs 9.628E‐04±2.706E‐05). Finally, positive subjects exhibited increased FW in the hippocampus (0.554±0.006 vs 0.511±0.010), isthmus cingulate (0.457±0.006 vs 0.430±0.009), medial temporal cortex (0.475±0.005 vs 0.453±0.008), and whole temporal cortex (0.497±0.005 vs 0.472±0.008). No significant regional differences were found in WM. For MMSE, MD (1.243E‐03±3.141E‐05 vs 1.140E‐03±2.419E‐05) and FW (0.561±0.009 vs 0.529±0.007) in the hippocampus were significantly elevated in the more impaired group.

**Conclusion:**

DTI was successfully implemented in phase 2 multi‐center PROSPECT‐ALZ study. Cross‐sectional analysis suggests that DTI measures can differentiate individuals based on global tau burden and cognition, presumably capturing differences in cortical microstructure. Future work will focus on multimodal evaluation of longitudinal changes in DTI, tau, and cognition.

